# Briquetting of Wastes from Pulp and Paper Industries by Using AOD Converter Slag as Binders for Application in Metallurgy

**DOI:** 10.3390/ma12182888

**Published:** 2019-09-06

**Authors:** Tova Jarnerud, Andrey V. Karasev, Pär G. Jönsson

**Affiliations:** KTH Royal Institute of Technology, 100 44 Stockholm, Sweden (A.V.K.) (P.G.J.)

**Keywords:** secondary raw materials, recirculation of wastes, metallurgical briquettes, resource saving

## Abstract

A number of carbon-rich (containing up to 47 wt% C) and lime-rich (containing up to 96 wt% of CaO-compounds) waste products from the pulp and paper industries can be used in iron and steel industry as fuels and slag formers for various metallurgical processes such as blast furnaces (BF), cupola furnaces (CF), argon oxygen decarburization (AOD) converters and electric arc furnaces (EAF). In most cases, these wastes consist of different size powders. In order to facilitate loading, transportation and charging of these powder wastes, briquetting is required. In this study, a pulverized AOD slag was tested as a binder component for briquetting of CaO-containing wastes (such as mesa, lime mud and fly ash) from pulp and paper industries. Moreover, mechanical testing of the possibilities for loading, transportation and unloading operations were done, specifically drop test trials were done for briquettes with different chemical compositions and treatments such as heating and storage. The results showed that an addition of 10–20% of AOD slag as a binder component followed by heat-treatment at 850 °C significantly improved the mechanical properties of the CaO-containing briquettes. An application of these briquettes will significantly reduce the consumption of natural resources (such as nature lime) in the metallurgical processes. Moreover, it can reduce the landfill area of wastes from pulp and paper industries, which is important from an environmental point-of-view.

## 1. Introduction

### 1.1. Wastes from Pulp and Paper Industry 

**Figure 1:**
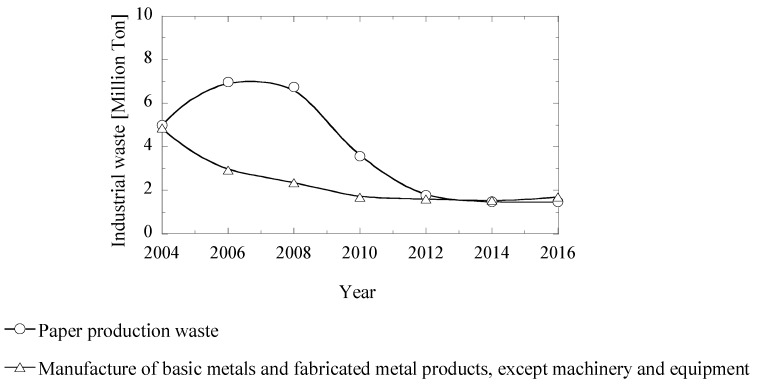
Annual amounts of wastes generated by pulp and paper industries and by metal industries in Sweden [2].

Sweden is one of the biggest producers of pulp and paper in Europe. For instance, 12.2 million metric tons of pulp and 10.3 million metric tons of paper were manufactured in 2017 [1]. However, it is well known that the pulp and paper industry also generates significant amounts of organic and inorganic wastes. As an example, almost 1.5 million metric tons of those wastes were obtained from the paper industry only in the year 2016 [2], as shown in Figure 1. For the most part, these wastes are usually kept in landfills, which can cause significant environmental problems due to chemical leaching and greenhouse gas emissions. Stronger regulations and requirements with respect to the environment, makes it more difficult and more expensive to dispose waste materials in landfills [3]. Moreover, waste materials kept in landfills represent non-sustainable solutions. 

A number of carbon-rich and/or lime-rich waste products from the pulp and paper industries can be used as a source of carbon and lime materials in iron and steel industries. This has the potential to considerably reduce the consumption and extraction of virgin natural raw materials used in metallurgical processes. Application of secondary materials (such as these wastes) can decrease the negative impact on the environment associated with mining of primary resources (such as disruptions of the landscape and effects on biodiversity) as well as to save the energy required for mining of natural resources. The natural materials cannot be fully replaced by these waste materials due to some undesired impurity elements, such as phosphorus, sulphur etc. These, in turn can deteriorate the quality of the produced iron and steel. To limit the amount of impurities, approximately 5–10% of the primary materials can be replaced by waste materials without having a significant effect on the quality of the final steel products. In this case, about 350,000 metric tons of wastes from the pulp and paper production can be treated and used in the iron and steel industries every year in Sweden [4].

**Figure 2:**
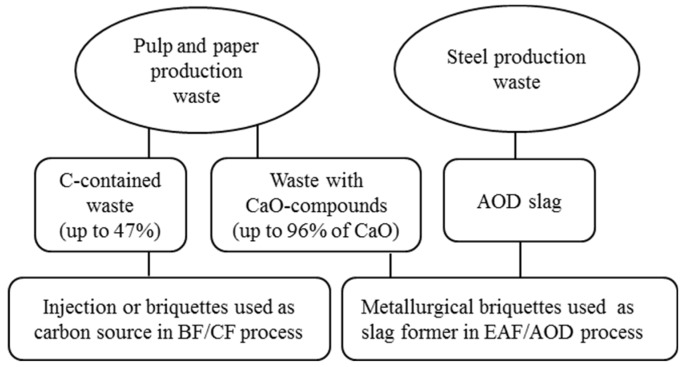
Application of wastes from pulp and paper industries in metallurgical processes.

The pulp and paper waste products can be divided into two main groups: 1) carbon-rich organic sludges and 2) lime-rich wastes, as illustrated in Figure 2. The carbon-rich wastes (contained up to ~47% of C) are called “mixed biosludge” and “fiber reject”. 

The lime-rich wastes (containing up to ~96% of CaO-compounds) are called “mesa”, “lime mud” and “fly ash”. Mesa comes from the unused boiling fluid in the preparation of white liquor [5], which consists of a sludge of insoluble calcium carbonate (CaCO_3_). Lime mud compounds are CaO-compounds obtained after calcination of mesa. Fly ash is a product of combustion of internal and external fuels (such as sludge from recycled paper and wood) in the pulp and paper production plants. However, there are still no clear and detailed evaluation with respect to applications of these wastes for briquetting, transportation as well as effective utilizations in various metallurgical processes to replace natural raw materials.

### 1.2. Steel Production Waste Materials

Almost two million metric tons of residuals were produced by the iron and steel industry in Sweden in 2015. Metallurgical slags accounted for ~70% of this (about 1,350,000 metric tons), and 6% (about 120,000 metric tons) was argon oxygen decarburization (AOD) slags. The characteristics, composition and properties differ significantly in the metallurgical slags depending on the process with which they were produced. To ensure an optimal use of waste products and thereby a reduced consumption of the required natural raw materials, the steel industry aim to substitute some of the traditional raw materials by wastes in various applications [6]. Overall, the re-use of steel slags depends on the influence of their chemical compositions on the properties.

Blast furnace slags are mostly used for road constructions, as raw materials for cement production or as cement-like binders [7]. However, swelling and disintegrating properties or leaching of metals can limit the use of some slags. Slags from the AOD converter typically have a ratio of lime and silica that promotes a formation of calcium silicates (2CaO·SiO_2_) during solidification. However, the β-form of this phase can expand during a phase inversion, when cooling through the temperature range of 400–500 °C [8]. This leads to a volumetric expansion of the material, which will break and cause the solid to fall into a fine powder. Therefore, the cooling rate of slags affects their textures, porosities and other physical properties [7]. This reduces the utilization scope of these slags and causes environmental deteriorations in the slag yard [9]. Also, a larger proportion of AOD and electric arc furnaces (EAF) slags from the production of high-alloyed steels is put to landfill compared to slags from production of low-alloyed steels. One of the reasons why slags from stainless steel production, having higher contents of Cr oxides, are less used for road constructions and cement production is that it is necessary to consider harmful effects caused by chromium leaching to the environment [10]. The binding properties of the AOD slags are a function of the specific surface (among other factors) and the fine powder promotes the binding abilities. When CaO and SiO_2_ are heated up to temperatures of around 700 °C, calcium silicate starts to form. This formation is promoted by small sizes of particles and close contacts [11]. The advantages of using AOD slags as binders in metallurgical briquettes includes that they are available materials in the steel plant, which have a well-known composition. When using this slag in metallurgical briquettes, the valuable alloying elements, such as chromium, lost to the slag during the steel production process are recirculated back into the steelmaking process. Furthermore, the usage of AOD slags will lead to a reduction of the amount of slag put to landfills in accordance with the pursuit of the circular economy concept. However, an effect of the chemical composition of AOD slags in metallurgical briquettes on their mechanical properties has not been investigated until now.

The aim of this study was to investigate the possibilities to use AOD slags as a binding agent in metallurgical briquettes obtained from different wastes from pulp and paper industries. Moreover, the influence of the recipes (contents of different base wastes and AOD slags), exposed time in air and heat treatment temperature on the mechanical properties of laboratory scale metallurgical briquettes obtained from the Ca-contained wastes from pulp and paper industry were studied.

## 2. Experimental

Waste materials obtained from pulp and paper industries such as mesa, lime mud and fly ash are fine powders, which should be briquetted for transportation and usage in metallurgical processes. Various types of binder components (such as bitumen, sorbitol, organic silica gel or Ca-stearate) can be used for briquetting. However, to avoid an increase of the hydrogen content in liquid steel after additions of briquettes and dehydration of the waste materials (or briquettes), a water free binder is required. In this study, a grinded AOD converter slag obtained during stainless steel production was tested as a binder component for briquetting of CaO-containing wastes. 

Depending on the specific production facility and even more on the specific production process and paper grade, the waste will have variations in the chemical composition. The chemical compositions of the CaO-containing waste powders used in this study are given in Table 1. It should be pointed out that mesa contains mostly CaCO_3_ (about 90%). To decrease the energy consumption and CO_2_ emissions by using these briquettes in metallurgical processes, the mesa need to be calcined at high temperatures. Lime mud, which was obtained after calcination of mesa, contains mostly CaO. The chemical composition of the material was made by ALS Scandinavia AB by using a Thermo finnigan element 1 Inductively Coupled Plasma Sector Field Mass Spectrometry (ICP-SFMS), (Thermo Scientific, Bremen, Germany) according to SS EN ISO 17294-1, 2 and the Environmental Protection Agency (EPA) method 200.8. The values from the analysis were recalculated to normalized values after Lost On Ignition (LOI) at 1000 °C, which is the mass of the volatiles lost during heating of material up to 1000 °C (organic matter, carbonates, moisture etc.). In addition to the main components, mesa and lime mud contains MgO (<1%) and the fly ash contains around 9% Al_2_O_3_, 3% MgO and <1% Fe_2_O_3_ and TiO_2_. However, these waste materials cannot fully replace the virgin lime during refining of iron and steels, due to the higher contents of P and S than those in suitable slag formers. 

One of the three Ca-rich waste products (mesa—M, lime mud—L and fly ash—F) as base material were mixed with various amounts of AOD slag and pressed into briquettes: (1) 100% base material (referred to as M100, L100 and F100), (2) 90% base material and 10% AOD slag (referred to as M90, L90 and F90), and (3) 80% base material and 20% AOD slag (referred to as M80, L80 and F80). The powder was placed in a stainless steel mould and pressed into a tablet shape briquette using a NIKE Hydraulics PHS70-700A hydraulic press. A 18 MPa pressure was applied for 10 s before the pressure was released and the briquette was collected from the mould. The briquettes were pressed one by one and the diameter of a briquette was 33 mm with a 18 mm thickness, weighing ~20 g each. The briquetting was carried out in air and at room temperature.

In order to evaluate the effect of the storage time in air on the impact strength of the briquettes, some briquettes were stored during 7, 20 and 35 days. The reason for these trials was to see if air storage can be used as a curing method and also to study how the briquettes can be stored for a future industrial use. 

To evaluate an effect of heat-treatment on the impact strength of the metallurgical briquettes, some samples were heated with a heating-rate of 10 °C/min and kept in the furnace for 30 min at 500 °C or for 60 min at 850 °C in air. Heating of briquettes up to 850 °C was done in two stages: (1) heating up to 500 °C and holding for 30 min, in order to avoid cracks due to gas formation inside the briquettes, and to allow gas and crystallized water to leave the briquettes before further heating and (2) heating up to 850 °C at 10 °C /min and holding for 60 min, to allow for a decomposition of CaCO_3_. To avoid a thermal stress and cracking of materials at fast cooling, the heat-treated briquettes were cooled down in furnace to room temperature. Then, all briquettes were kept in a desiccator until the drop test trials were carried out. For each combination of chemical composition (recipe) and set of test and treatment conditions, a minimum of three briquette samples (S1, S2 and S3) having the same composition and treatment conditions were produced and tested. Totally, 90 briquettes were prepared and tested in this study.

Drop tests were used for comparison of the impact strength of different briquettes. Each briquette was dropped with the flat face down from a 1.0 m height onto a steel plate. The largest piece of a briquette was weighted using an A&D ER-180A Electric balance (retained weight—RW) and used for the following drop test after cleaning of the steel plate. Since the small pieces (or dust) of broken briquettes cannot be technologically used for charging in some metallurgical processes the minimum possible weight loss of a briquette was evaluated by weighing the biggest briquette piece before and after drop test. The drop test was repeated until the retained part of the briquette was less than 1% of the initial mass of the briquette or after a maximum of 15 drops. In this study, it was assumed that the briquette has to withstand 5 to 7 drops in industrial conditions during loading, transportation and unloading without causing a fast destruction into powder.

The effect of the chemical composition recipes and heat treatment of briquettes on their mechanical properties were evaluated by detailed investigations of the base materials and briquettes based on fly ash. The specimens of materials for scanning electron microscopy (SEM) investigations were cold mounted in EpoFix, polished without water, and covered with Au/Pd. Estimations of structures and compositions of main compounds in powders of fly ash and AOD-slag and in briquette pieces before and after heat treatments were done by using a Hitachi S-3700N (Tokyo, Japan) SEM equipped with a Bruker AXS XFlash Detector 4010 (MA, USA) in combination with energy dispersive spectroscopy (EDS). The SEM imaging was done using a 10–11 mm working distance and a 15 kV acceleration voltage. The composition of each main compound in different materials was determined based on 5 to 8 measurements by using an “area” mode analysis and based on 10–15 measurements using point analysis in different zones of samples.

## 3. Results and Discussion

**Figure 3:**
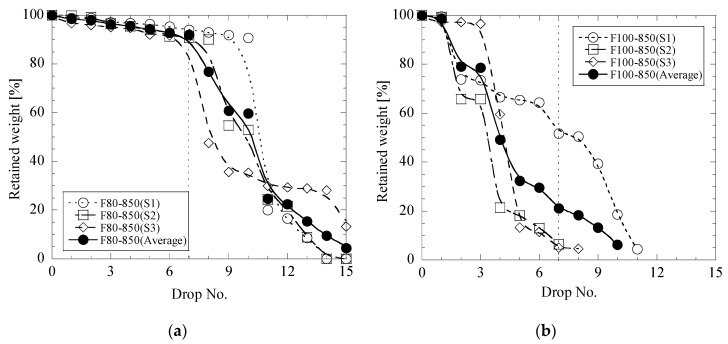
Relationships between retained weight of briquettes and drop number in drop test trials for briquettes of F80-850 (**a**) and F100-850 (**b**).

In this study, three samples (S1, S2 and S3) for each type of briquettes were drop tested for evaluation of the statistically relevant average value of the impact strength. It was found that the standard deviations of retained weight obtained after drop No.7 (σ_7_) of these three samples can vary in significant ranges for the same type of briquettes. Figure 3a,b shows some trials which have good (σ_7_ < 5%) and bad (σ_7_ > 15%) repeatability, respectively. The presented briquettes had the σ_7_ values of 2 and 28%. It is interesting to point out that the scatter of the drop test results increased for briquettes which were heat treated at 850 °C in the following order: from mesa (σ_7_ is 3~9%), from lime mud (σ_7_ is 9~13%) and from fly ash (σ_7_ is 2~28%). Therefore, the following results of drop test trials for different types of briquettes are presented as average values for the three briquette samples.

The results show that most of the briquettes cannot be transported and used in metallurgical processes without any follow-up treatment. All briquettes based on mesa and lime mud were almost completely destroyed after 2–6 drops. The fly ash briquettes F100, F90 and F80 retained only 33, 17 and 3% of their initial weight after seven drops, respectively.

**Figure 4:**
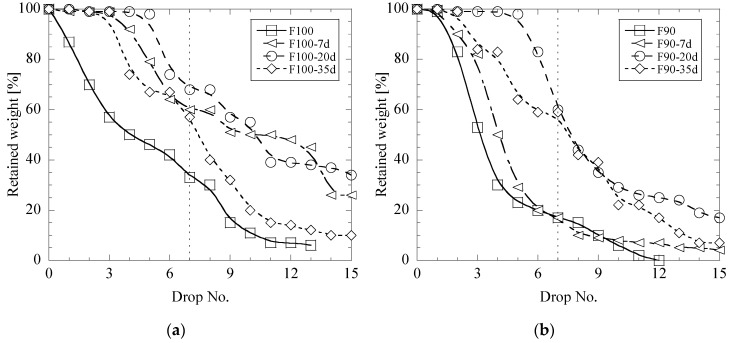
Effect of storage of briquettes F100 (**a**) and F90 (**b**) in air on the retained weight during drop test trials.

Storage in air of the briquettes based on mesa and lime mud during 7, 20 and 35 days did not improve their impact strength. All these briquettes were destroyed during storage (as for lime mud briquettes) or after 6 drop tests (as for mesa briquettes). Figure 4 shows relationships between retained weights of briquettes and the drop numbers in drop test trials for fly ash briquettes (F100 and F90) for different storage times of 7, 20 and 35 days. It can be seen that the storage of F100 and F90 briquettes in air during 20–35 days can significantly increase the impact strength of these briquettes. For instance, the retained weight of briquette pieces after 7th drop test is 60–68% and 57–59% after holding during 20 and 35 days, respectively. These values are ~2–3 times larger compared to those for F100 and F90 samples, which have not been stored before being tested.

**Figure 5:**
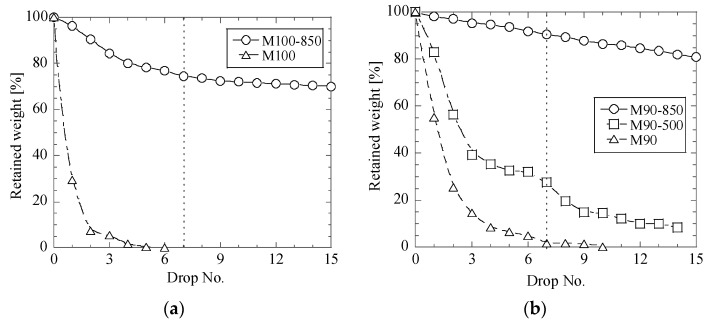

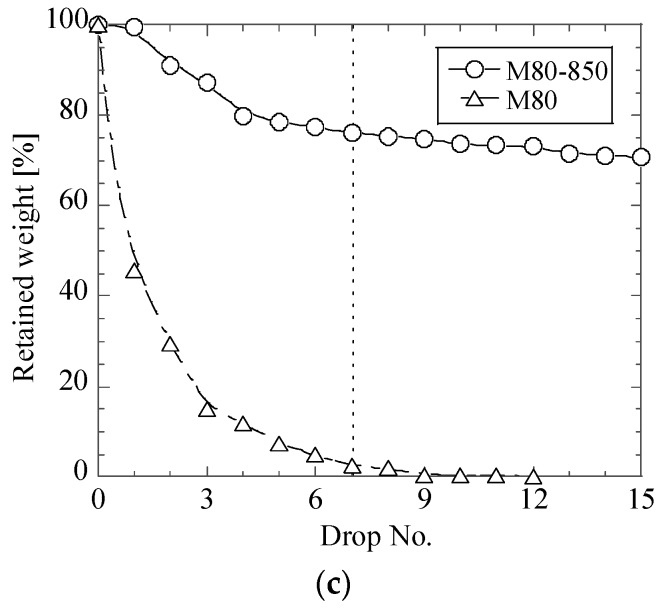
Relationships between retained weight of briquettes M100 (**a**), M90 (**b**) and M80 (**c**) and drop number in drop test trials for mesa briquettes before and after heat treatment at 500 °C and 850 °C.

**Figure 6:**
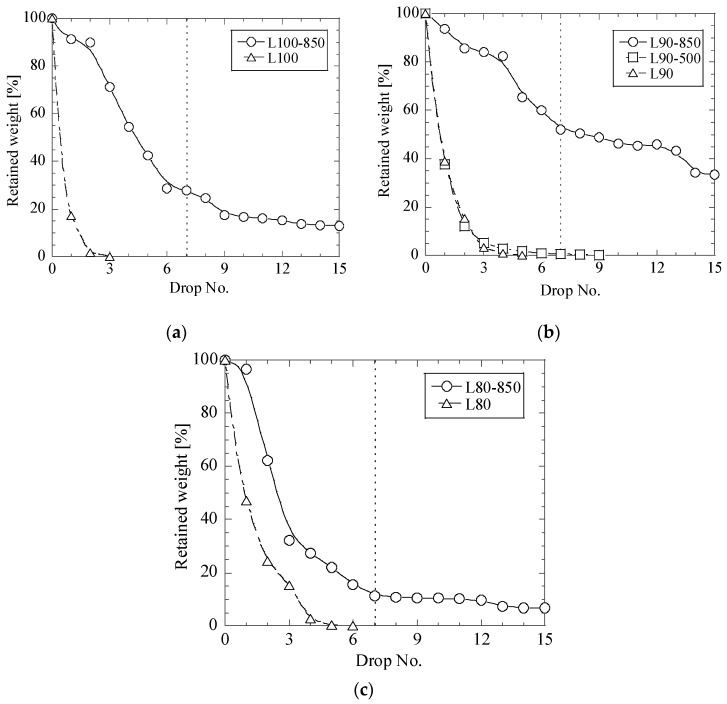
Relationships between retained weight of briquettes L100 (**a**), L90 (**b**) and L80 (**c**) and drop number in drop test trials for different lime mud briquettes before and after heat treatments at 500 °C and 850 °C.

**Figure 7:**
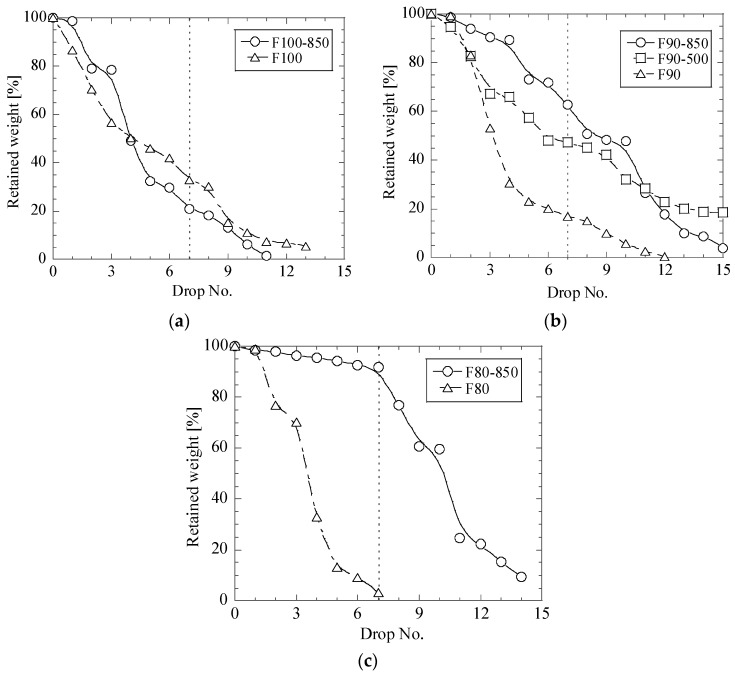
Relationships between retained weight of briquettes F100 (**a**), F90 (**b**) and F80 (**c**) and drop number in drop test trials for different fly ash briquettes before and after heat treatment at 500 °C and 850 °C.

It was revealed in this study that the heat treatment of the CaO-contained briquettes can strongly increase the impact strength. Figure 5, Figure 6 and Figure 7 show the relationships between the retained weight of briquettes and drop numbers in drop test trials for different briquettes before and after heat treatments at 500 °C and 850 °C.

It can be seen in Figure 5 that the heat treatment at 500 °C and 850 °C increased the retained weight of briquettes after the seventh drop test from 1–2% before heat treatment up to 28% for the M90-500 briquettes and up to 74–91% for the M100-850, M90-850 and M80-850 briquettes. The largest improvement of the impact strength for the mesa briquettes was obtained for M90-850 briquettes (Figure 5b, RW ~ 91%).

Heat treatment at 500 °C of briquettes based on lime mud (L90-500) does not improve the impact strength, as shown in Figure 6b. However, the RW values can be increased slightly after heat treatment at 850 °C. Specifically, up to 28% in L100-850, 52% in L90-850 and 11% in L80-850 briquettes. The best impact strength for the lime mud briquettes was obtained for the L90-850 briquettes. 

It can be seen in Figure 7 that the heat treatment at 500 °C of fly ash briquettes can significantly increase the retained weight of briquettes after the 7th drop test from 17% before a heat treatment (F90) up to 47% in F90-500 briquettes. The heat treatment at 850 °C of most fly ash briquettes increases the retained weight of briquettes by up to 63% in F90-850 and 92% in F80-850 briquettes. However, the impact strength for the F100-850 briquettes does not improve after a heat treatment at 850 °C (RW ~ 21%). Therefore, the heat-treatment of pure fly ash briquettes at 850 °C cannot improve the impact strength. However, an addition of 10% of AOD slag into metallurgical briquettes can significantly increase the strengths for the heat-treated briquettes, as are the case for mesa and lime mud briquettes. The largest improvement of the impact strength for the fly ash briquettes was obtained for F80-850 briquettes containing 20% of AOD slag (Figure 7c, RW ~ 92%).

**Figure 8:**
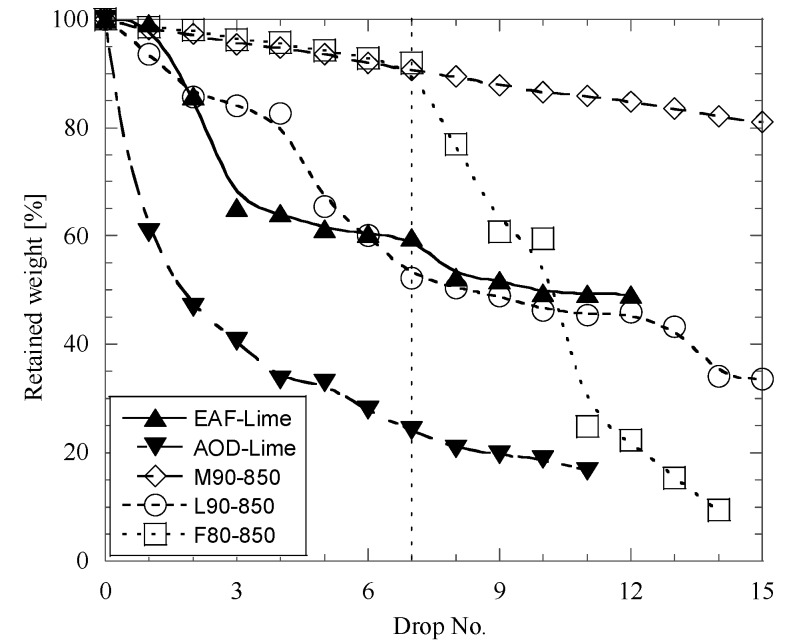
Comparison of drop test results for the best metallurgical briquettes (M90-850, L90-850 and F80-850) and lime lumps for electric arc furnaces (EAF) and argon oxygen decarburization (AOD) converter.

A comparison of drop test results obtained for the best metallurgical briquettes, which were prepared by using different wastes from pulp and paper industries and AOD slag, and for typical lime lumps used for usual steelmaking in EAF and AOD converter, is shown in Figure 8. It can be seen that the retained weight of briquettes after the seventh drop test for M90-850 and F80-850 briquettes is much larger than that for AOD-lime and EAF-lime [12]. Moreover, the L90-850 briquettes and EAF-lime lumps have similar mechanical properties. 

Finally, it should be noted that all the metallurgical briquettes based on mesa (M100-850, M90-850 and M80-850) have significantly better impact strengths after heat treatment at 850 °C compared to the briquettes based on fly ash and lime mud, as shown in Figure 5, Figure 6 and Figure 7. This may be explained as follows: though water was not added during briquetting, the mesa powder, which was used for preparation of metallurgical briquettes, contained slightly more moisture than lime mud and fly. In this case, water promotes the formation of a calcium silicate hydrate glue (3CaO·2SiO_2_·3H_2_O), which increases the binding effects. Moreover, when CaO and SiO_2_ are heated up to temperatures of around 700 °C in a strongly alkaline environment, the insoluble SiO_2_ becomes soluble, which leads to that compounds of Dicalcium silicate, 2CaO·SiO_2_ (C_2_S, Belite) starts to form. This can also improve the binding effect. In this case, a better binding in the mesa-based briquettes can be obtained during heat treatment of the briquettes at 850 °C, as was confirmed by the obtained results. Also, as in all dry chemical reactions, the reaction is promoted by a fine size and a close contact of particles in briquettes [11].

**Figure 9:**
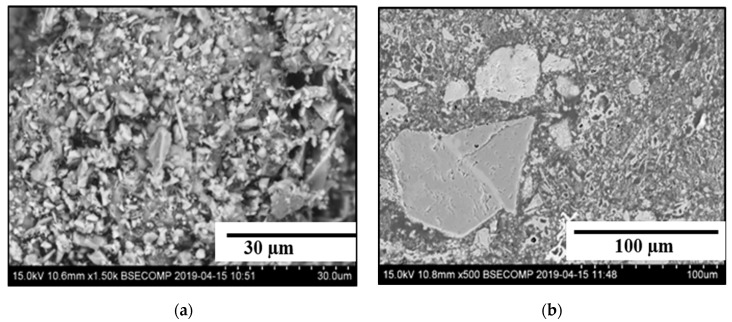
Typical scanning electron microscopy (SEM) images of small particles (**a**) and large blocks (**b**) in investigated powders and briquettes.

The effect of the chemical composition of recipes and heat treatment of briquettes on their mechanical properties were evaluated by detailed investigations of the studied materials and briquettes based on fly ash. Estimations of the structures and compositions of main compounds in powders of fly ash and AOD slag and in briquettes before and after heat treatments were done by using SEM in combination with EDS. It was found that all investigated materials (powders and briquettes) contained small size particles (0.5–10 µm) and large size blocks having different sizes from 10 up to 500 µm, as shown in Figure 9.

According to compositions, all compounds in the investigated materials were classified into three main types. Compounds of type I contain mostly CaO (75–99%) and some amount of SiO_2_ (0–13%), Al_2_O_3_ (0–11%), MgO (0–4%), CaS (0–4%) and Na_2_O + K_2_O (0–2%). Compounds of type II contain 50–74% of CaO, 15–43% of SiO_2_, 0–15% of Al_2_O_3_, 0–10% of MgO, 0–2% of CaS and 0–0.4% of Na_2_O + K_2_O. Type III compounds contain 34–58% of CaO, 5–42% of SiO_2_, 6–39% of Al_2_O_3_, 1–17% of MgO, 0–1% of CaS and 0–4% of Na_2_O + K_2_O. Also, the average compositions of different compounds in some material samples are given in Table 2. 

**Figure 10:**
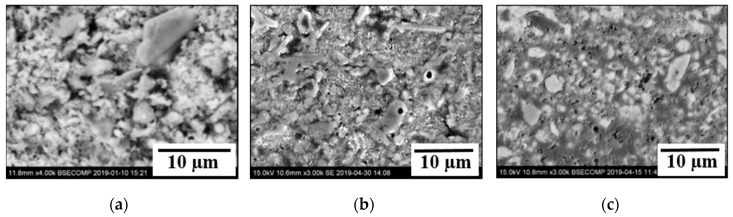
Typical SEM images of small particles in fly ash powder (**a**) and in F80 briquettes before (**b**) and after (**c**) heat treatment at 850 °C.

It should be pointed out that all three types of compounds were observed in the base materials (fly ash and AOD-slag powders) and in all investigated briquettes. Furthermore, different size blocks and small particles from the same compound types have similar compositions. The determined chemical compositions of different compounds before and after heat treatments do not reveal clear relationships between the chemical compositions and the heat treatment. This is most likely due to wide composition variations of these compounds. However, visual investigations of small particles in briquettes after heat treatments at 850 °C showed that most of the particles were sintered despite their different compositions. As shown in Figure 10, the separate particles in powders (Figure 10a) and briquettes (Figure 10b) before a treatment were sintered after a completed heat treatment (Figure 10c). This can explain the better drop test results for heat treated briquettes (the retained weight after 7 drops were 3% for F80 briquettes, and 92% for F80-850 briquettes).

The significantly improved (4–30 times) mechanical properties of F90-850 and F80-850 briquettes in comparison to the F90 and F80 briquettes can be explained by sintering of small particles of Type II and Type III at 850 °C, as was discussed above. It was found that the chemical compositions of Type II particles sintered together with particles of Type III are similar to the composition of Belite (containing 63.5% of CaO, 31.5% of SiO_2_, 2.1% of Al_2_O_3_, 0.5% of MgO and ~1.0% of Na_2_O + K_2_O). This is an important industrial mineral used as a binder component in Portland cement, which is used in manufacturing and in pellets [13].

## 4. Conclusions

Metallurgical briquettes were prepared in a laboratory scale by using different CaO-containing waste materials (mesa, lime mud and fly ash) from pulp and paper industries and pulverized AOD converter slags as binders. The mechanical properties of the briquettes were studied by using drop testing for different chemical compositions, holding times in air and heat treatment conditions. The drop test results obtained for the best metallurgical briquettes were compared to those for conventional lime lumps, which are used for steelmaking in EAF and AOD reactors. The following specific conclusions can be made based on the results of this study:

- An addition of AOD-slag (10–20%) as a binding agent in all briquette types without using an additional heat-treatment does not improve the impact strength.
- Mesa and lime mud briquettes cannot be stored in an open atmosphere without falling apart. However, fly ash briquettes which are stored for 7, 20 and 35 days are about 2–3.5 times stronger in most trials than those briquettes which did not get additional treatments.
- Heat-treatment of briquettes at 500 °C both cannot improve (L90-500) the strength, or can improve (M90 from 2% up to 28% for M90-500, and F90 from 17% up to 47% for F90-500), but not as much as when using a heat-treatment at 850 °C.
- For 100% fly ash briquettes, a heat-treatment at 850 °C does not improve the impact strength. However, for 100% mesa (M100 from 0% up to 74% for M100-850) and 100% lime mud (L100 from 0% up to 28% for L100-850) briquettes, the impact strength is improved after a heat treatment.
- Addition of 10–20% of AOD converter slag in combination with a heat-treatment at 850 °C can significantly improve (from 0% up to 92% retained weight after seven drops) the impact strength of the metallurgical briquettes based on CaO-contained wastes from pulp and paper industries. The best briquettes have much larger (M90-850 and F80-850) or similar (L90-850) mechanical properties as conventional lime lumps (M90-850 retained 91%, F80-850 retained 92%, L90-850 retained 52%, EAF lime lumps retained 60%, AOD lime lumps retained 23% of its initial weight after seven drops) for steelmaking in EAF and AOD converter.

With this method, waste products from two industrial sectors can be treated and utilized as secondary raw materials and reduce the amount of waste put to landfill as well as the extraction of natural resources. Furthermore, it can lower greenhouse gas emissions and it can save money for both the pulp and paper and iron and steel producers.

## Figures and Tables

**Table 1:** Contents of main components (in wt%) of lime rich waste materials used in metallurgical briquettes.

Materials	CaO	Na_2_O	P	SiO_2_	K_2_O	S	Balance	LOI 1000 °C
Mesa *	95.9	1.5	0.4	0.2	0.2	0.1	1.7	40.6
Lime mud *	95.9	1.5	0.4	0.1	0.1	0.2	1.8	6.1
Fly ash *	63.2	0.6	0.1	21.0	0.5	0.4	14.2	6.6

* Composition of materials after calcination at 1000 °C.

**Table 2:** Average contents (in mass%) and standard deviations of main components in different types of compounds in analyzed material samples.

Sample	Compound	MgO	Al_2_O_3_	SiO_2_	CaO	CaS	Na_2_O + K_2_O
Fly ash	Total	2.2 ± 0.6	5.5 ± 1.4	14.2 ± 4.9	76.6 ± 2.8	0.9 ± 0.4	0.7 ± 0.5
-	I	2.0 ± 0.8	3.6 ± 1.4	8.5 ± 3.8	83.6 ± 5.7	1.6 ± 1.3	0.7 ± 0.7
-	II	3.7 ± 3.8	3.1 ± 2.8	27.6 ± 5.1	64.7 ± 2.6	0.8 ± 0.3	0.1 ± 0.2
-	III	6.6 ± 7.1	18.6 ± 7.2	32.3 ± 6.0	40.1 ± 6.0	0.1 ± 0.2	2.3 ± 1.5
AOD-slag	Total	5.2 ± 0.5	13.1 ± 3.7	23.0 ± 2.5	58.4 ± 1.5	0.4 ± 0.3	0
-	I	0.9 ± 0.6	10.9 ± 2.1	12.4 ± 1.9	74.7 ± 4.6	0.3 ± 0.3	0
-	II	6.5 ± 3.2	4.5 ± 3.1	33.7 ± 4.4	55.1 ± 4.3	0.3 ± 0.7	0
-	III	3.4 ± 2.1	24.5 ± 2.0	17.3 ± 1.9	53.5 ± 1.5	0.9 ± 0.6	0
F80	Total	3.1 ± 0.4	7.1 ± 1.1	19.6 ± 4.2	70.0 ± 2.6	0	0.3 ± 0.1
-	I	1.1 ± 0.5	0.6 ± 1.0	1.4 ± 2.0	96.9 ± 3.1	0	0
-	II	1.7 ± 1.4	10.6 ± 4.5	23.4 ± 5.5	64.1 ± 5.3	0	0.2 ± 0.5
-	III	9.4 ± 6.8	11.5 ± 8.3	30.0 ± 9.3	47.5 ± 9.2	0	1.6 ± 1.8
F80-850	Total	3.0 ± 0.3	17.5 ± 15.4	17.6 ± 7.0	61.4 ± 12.4	0	0.5 ± 0.6
-	I	1.8 ± 1.2	2.8 ± 3.5	6.0 ± 6.7	89.4 ± 10.7	0	0.1 ± 0.1
-	II	2.9 ± 3.6	4.5 ± 5.6	27.6 ± 5.0	64.8 ± 4.6	0	0.2 ± 0.3
-	III	2.5 ± 2.7	24.5 ± 11.2	21.9 ± 12.9	48.4 ± 7.4	0	1.5 ± 1.6
